# Trends in handwashing behaviours for COVID-19 prevention: Longitudinal evidence from online surveys in 10 sub-Saharan African countries

**DOI:** 10.1371/journal.pgph.0000049

**Published:** 2021-11-12

**Authors:** Bolanle Olapeju, Zoé Mistrale Hendrickson, Joseph G. Rosen, Dominick Shattuck, J. Douglas Storey, Susan Krenn, Marla Shaivitz, Elizabeth Serlemitsos, Tuo-Yen Tseng, Samantha W. Tsang, Rajiv N. Rimal, Stella Babalola

**Affiliations:** 1 Department of Health, Behavior and Society, Johns Hopkins Bloomberg School of Public Health, Baltimore, Maryland, United States of America; 2 Johns Hopkins Center for Communication Programs, Baltimore, Maryland, United States of America; 3 Department of International Health, Johns Hopkins Bloomberg School of Public Health, Baltimore, Maryland, United States of America; Pennsylvania State University, UNITED STATES

## Abstract

Handwashing is essential for respiratory virus prevention, but uptake of handwashing in the context of the SARS-CoV-2 pandemic remains under-explored. This study examines trends in and determinants of handwashing practices for COVID-19 prevention in 10 countries in West, East, and Southern Africa. Data are derived from an online global Facebook survey assessing COVID-19 knowledge, attitudes, and practices, fielded in July (Round 1) and November 2020 (Round 2). Adults ≥18 years (*N* = 29,964) were asked if they practiced handwashing with soap and water in the past week to prevent COVID-19. Design-corrected F-statistics compared knowledge and practice of handwashing, at country and regional levels, between survey rounds. A country-level fixed-effects logistic regression model then identified socio-demographic and ideational correlates of handwashing at Round 2. Most participants were >30 years-old, men, post-secondary educated, and urban residents. Between survey rounds, handwashing prevalence declined significantly across regions and in each country, from a 14% decline (Δ84%–70%) in Tanzania to a 3% decline (Δ92%–89%) in South Africa. Handwashing was higher among participants aged >30 years (Adjusted Odds Ratio [aOR] = 1.25, 95% confidence interval [95%CI]: 1.15–1.35) and with post-secondary education (aOR = 1.62, 95%CI: 1.49–1.77) but lower among men (aOR = 0.71, 95%CI: 0.64–0.78). Ideational factors associated with handwashing included perceived effectiveness of handwashing (aOR = 2.17, 95%CI: 2.00–2.36), knowing someone diagnosed with COVID-19 (aOR = 1.28, 95%CI: 1.18–1.40), and perceived importance of personal action for COVID-19 prevention (aOR = 2.93; 95%CI: 2.60–3.31). Adjusting for socio-demographic and ideational factors, country-level marginal probabilities of handwashing ranged from 67% in Tanzania to 91% in South Africa in Round 2. COVID-19 prevention messages should stress the importance of handwashing, coupled with mask use and physical distancing, for mitigating respiratory disease transmission. Behaviour change communications should be sensitive to resource heterogeneities in African countries, which shape opportunities for sustainable handwashing behaviours.

## Introduction

The SARS-CoV-2 (COVID-19) pandemic has affected the health and well-being of people around the world. As of August 16, 2021, over 207 million COVID-19 cases have been confirmed–with over 4.3 million deaths recorded across continents [1]. In sub-Saharan Africa, prevalence of COVID-19 cases varies across settings. In South Africa, where an identified SARS-CoV-2 variant of concern (B.1.351) has posed additional challenges for mitigating transmission (Centers for Disease Control and Prevention) [2], over 1.5 million cases have been confirmed [1]. While considerable investment in the development and roll out of vaccines for COVID-19 has been made, behavioural prevention will continue to be critical to reducing COVID-related morbidity and mortality. Handwashing is an important strategy for minimizing transmission of COVID-19 and other respiratory viruses like the 2009 H1N1 [3, 4] as well as diarrheal diseases and outbreaks of viral hemorrhagic fevers like Ebola [5, 6].

Individuals’ ability to practice this important COVID-19 preventive behaviour is influenced by factors operating at multiple socio-ecological levels, ranging from individual to interpersonal to community or structural factors. At the community or structural level, availability of handwashing stations, equipped with water and soap, is critical. A recent study estimated that, prior to the COVID-19 pandemic, more than half of people living in sub-Saharan Africa did not have access to a handwashing station [7, 8]. The percentage of households with an observed place for handwashing varied dramatically across settings [7, 8]. Those living in rural areas and those from lower wealth quintiles, for example, often have less access to a place for handwashing than those in urban areas and higher wealth quintiles [9, 10]. Even when handwashing stations are available, an analysis of data from 16 countries in Sub-Saharan Africa found that only 34% of those with a place to wash their hands had water and soap [9, 10]. Water insecurity further exacerbates these challenges [11]. Together, these structural factors affect community members as well as healthcare workers, who also often face challenges to accessing handwashing stations or lack sufficient access to the water and soap needed to wash one’s hands sufficiently to prevent COVID-19 transmission [12–14]. In settings where water and handwashing stations are available, however, individual and interpersonal barriers to handwashing persist. In the Democratic Republic of the Congo in the early phase of the COVID-19 pandemic, researchers observed that while there were handwashing facilities in public markets, they were not being used [15]. Ideation offers a useful theoretical framework through which to understand these barriers and identify determinants of handwashing practices. Ideation posits that individuals’ behaviours are influenced by their beliefs, feelings, or interactions, and that behaviours can change if these cognitive, emotional, or social “ideational” factors change [16, 17]. Researchers have identified numerous ideational factors that influence handwashing practices [18–20]. The risk, attitudes, norms, ability, and self-regulation (RANAS) framework [21] is one approach used to describe how handwashing is influenced by numerous cognitive ideational factors like individuals’ knowledge about the importance of handwashing for preventing disease transmission, perceptions about the effectiveness of handwashing in preventing transmission, or perceived susceptibility to illnesses like COVID-19. Emotional ideational factors include emotional responses like optimism, self-efficacy, or guilt that can influence handwashing practices [22–24]. Social ideational factors include perceived social influence or pressure to hand-wash and perceptions of what others think about handwashing (i.e., injunctive norms) or what others are doing (i.e., descriptive norms) [21]. Collectively, these ideational factors, coupled with aspects of the built environment, influence individuals’ ability and motivation to practice a COVID-19 prevention behaviour like handwashing.

As the practice of key preventive behaviours like handwashing continue to play critical roles in mitigating COVID-19 transmission, greater attention to the prevalence of handwashing practices, and understanding the determinants of handwashing in sub-Saharan Africa, during the COVID-19 pandemic is necessary. While numerous commentaries and opinion pieces have identified the importance of handwashing [25–27], there is a dearth of empirical evidence on handwashing practices in the context of the COVID-19 pandemic. To fill this gap and inform ongoing and future COVID-19 prevention efforts, this article examines trends in and determinants of handwashing practices for COVID-19 prevention in West, East, and Southern Africa.

## Materials and methods

Data are derived from online serial cross-sectional surveys assessing COVID-19 knowledge, attitudes, and practices; survey methods have been described previously [28]. Briefly, between July 6^th^ and 20^th^, 2020, adults 18 years and older from 67 countries received a notification at the top of their Facebook newsfeeds to complete an online survey. Participants responded to questions measuring socio-demographics, exposure to media and information pertaining to COVID-19, knowledge of COVID-19 prevention and treatment, awareness of people diagnosed with COVID-19, use of healthcare services during the pandemic, and COVID-19 vaccination intentions. Participants were then randomized to receive four of nine additional question blocks measuring COVID-related attitudes, behaviours, and practices. No participants were shown all nine additional question blocks.

**Fig 1:**
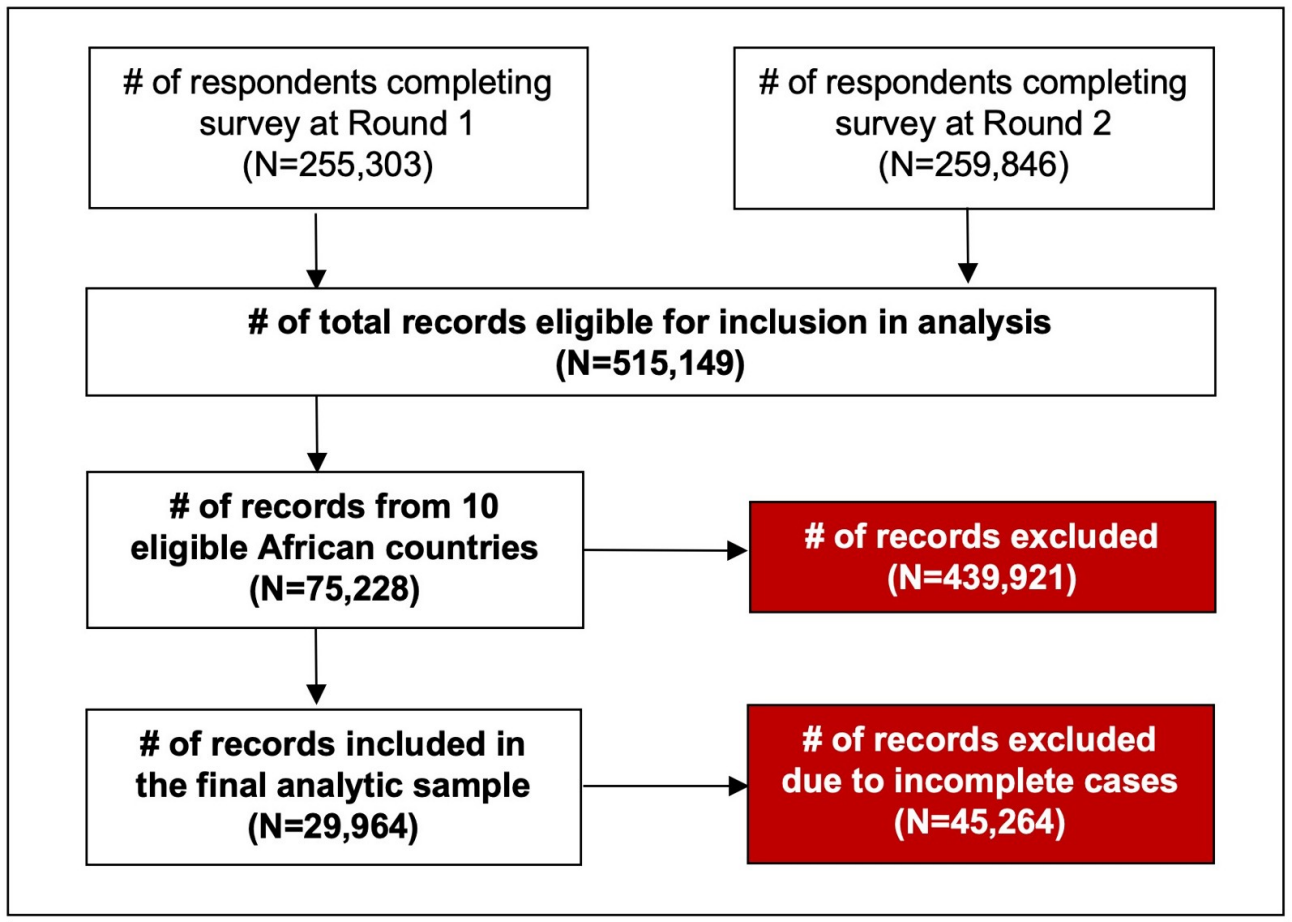
Inclusion schema for the current study.

The survey was re-administered in sequential two-week intervals in 23 of the 67 countries throughout 2020, producing serial cross-sectional samples of adult Facebook users. From November 9th to 25th, 2020 (corresponding to the 10^th^ data collection wave), the survey was launched again in the 67 countries included in the initial wave of data collection (July 2020). Only data from the following 10 African countries sampled in the first (Round 1) and tenth (Round 2) waves were included in this analysis: Angola, Côte d’Ivoire, Ghana, Kenya, Mozambique, Nigeria, Senegal, South Africa, Tanzania, and Uganda. Inclusion schema for this analysis is illustrated in Fig 1.

### Measures

The primary outcome was handwashing, which was measured dichotomously (“yes” vs. “no”) from a single item asking participants whether they had practiced handwashing with soap and water in the past week to prevent COVID-19 infection.

Socio-demographic factors included age group in years, measured ordinally (<20, 20–30, 31–40, 41–50, 51–60, 61–70, 71–80, or ≥80) but collapsed into a dichotomous variable (≤30 vs. >30 years) to distinguish youth and younger adults from older adults; gender (men or women); educational attainment, comparing those with college/university education or higher (post-secondary) to those with secondary school-level education or less; and residence in an urban (“city” or “town”) or rural (“village or rural area”) locality.

Other independent variables included theorized cognitive, emotional, and social ideational factors associated with handwashing for COVID-19 prevention. First, cognitive ideational factors included individuals’ self-reported beliefs about their own health status, perceived effectiveness of handwashing, and attitudes about handwashing. Self-reported health status compared participants who reported being in “excellent” or “very good” health to those who reported “good”, “fair”, or “poor” health. Perceived effectiveness of handwashing was measured by comparing participants who endorsed handwashing as a very or extremely effective prevention strategy for COVID-19 to participants who did not (“moderately”, “slightly”, or “not at all” effective). Attitudes related to personal action were assessed by asking participants: “How important is it for you to take actions to prevent the spread of COVID-19?”. Responses were measured on a 5-point Likert scale (“extremely important” to “not important at all”) and collapsed into dichotomous covariates, comparing participants who perceived personal action to be “extremely” or “very” important to prevent COVID-19 to those who perceived them as “moderately”, “slightly”, or “not at all” important. Second, knowing someone who tested positive with COVID-19 was used as a proxy for emotional response related to COVID-19, an emotional ideational factor. Participants were also asked if they knew someone who tested positive to COVID-19 (“yes” vs. “no” / “refused to answer”). Third, community-level perceived norms surrounding personal action, a social ideational factor, was measured by asking participants: “How important do other people in your community think it is to take actions to prevent the spread of COVID-19?”. As described above, responses were measured using a 5-point Likert scale and dichotomized to compare those reporting “extremely” or “very” important to those who said “moderately”, “slightly”, or “not at all” important.

### Analysis

Data were managed and analyzed in Stata/IC 15.1 (StataCorp, College Station, Texas). Because of the question block randomization approach utilized in the survey, missing responses were frequent. Listwise deletion (complete case analysis) was used to exclude any participants with missing observations for the primary outcome or any independent variables included in multivariable analysis. To evaluate the degree of selection bias introduced into the analysis by excluding missing observations, sample characteristics (i.e., covariate distributions and chi-square tests of association) at Round 1 and 2, respectively, were compared between complete cases retained in the analytic sample and excluded cases.

Descriptive sample statistics were first calculated and compared over survey rounds. All descriptive analyses were weighted using an analysis weight estimated from the probability of non-response and differences in age/sex distributions between sampled Facebook users and national populations within each country. Statistically significant (*p*<0.05) differences in handwashing and other independent variables between survey waves were quantified using a design-corrected (i.e., weighted) F-statistic. Within each country, the proportion of participants reporting handwashing and perceived effectiveness of handwashing for COVID-19 prevention, respectively, were compared over survey rounds using a design-corrected F-statistic.

To identify ideational correlates of handwashing and to estimate the probability of handwashing conditioned on country of residence, a multivariable binomial logistic model regressed self-reported handwashing onto socio-demographics and ideational factors at Round 2 only. Country of residence was also included in the model as a fixed effect. Adjusted marginal probabilities of handwashing by country in Round 2 were estimated from transformed logistic regression coefficients (Adjusted Odds Ratios [aOR] and 95% Confidence Intervals [95%CI]).

### Ethical statement

The Committee on the Use of Humans as Experimental Subjects at the Massachusetts Institute of Technology (Cambridge, MA) reviewed and approved the study protocol (#E-2294). Participants provided signed electronic consent prior to completing the online survey. All study protocols conformed to the human subjects’ research principles outlined in the Declaration of Helsinki.

## Results

Table 1 presents country-specific sample sizes by survey round. A total of 9,876 participants responded to the specific survey questions in Round 1 and 20,088 in Round 2. Across countries, the second round of the survey aligned with increasing cumulative growth of COVID-19 cases.

**Table 1:** Country-specific survey sample sizes and cumulative national COVID-19 infections per capita, by survey round (*N* = 29,964).

	July 6–20, 2020		November 9–25, 2020	
	Round 1 (*n* = 9,876)	Cumulative COVID-19 cases (per 100,000)*	Round 2 (*n* = 20,088)	Cumulative COVID-19 cases (per 100,000)*
**West Africa**				
Côte d’Ivoire	675 (17.6%)	54.3	2,113 (27.7%)	80.2
Ghana	855 (22.3%)	91.5	2,190 (28.7%)	164.9
Nigeria	1,673 (43.6%)	18.1	2,161 (28.3%)	32.4
Senegal	635 (16.5%)	53.4	1,166 (15.3%)	95.1
**East Africa**				
Kenya	1,135 (37.8%)	25.6	2,149 (36.4%)	147.5
Tanzania	725 (24.1%)	1.0	1,597 (27.0%)	1.0
Uganda	1,143 (38.1%)	2.3	2,165 (36.6%)	41.3
**Southern Africa**				
Angola	945 (31.1%)	2.3	1,888 (28.8%)	45.1
Mozambique	997 (32.9%)	4.8	2,206 (33.7%)	49.0
South Africa	1,093 (36.0%)	630.09	2,453 (37.5%)	1307.6

* Estimated using reported COVID-19 cases from each country through the end date of survey implementation at each round (Round 1: July 20, 2020; Round 2: November 25, 2020), Source: Johns Hopkins University & Medicine Coronavirus Resource Center.

Tables 2 and 3 summarize results of sensitivity analyses, comparing complete cases (i.e., participants with non-missing responses for covariates of interest) to excluded cases with missing data. Across rounds, complete cases were significantly more likely than excluded cases to report handwashing in the past week for COVID-19 prevention (Round 1: 90.3% vs. 85.2%, *p*<0.001; Round 2: 83.0% vs. 76.9%, *p*<0.001) and to have post-secondary education (Round 1: 77.4% vs. 72.7%, *p*<0.001; Round 2: 71.7% vs. 64.7%, *p*<0.001), although quantitative differences in point estimates were negligible. In Round 2 only, complete cases were significantly more likely to report knowing someone diagnosed with COVID-19 compared to excluded cases (44.9% vs. 38.2%, *p*<0.001). Other statistically significant (*p*<0.05) differences identified between complete and excluded cases were numerically negligible.

**Table 2:** Unweighted descriptive sample statistics comparing complete cases (included in analysis) to those excluded from analysis due to missingness in Round 1.

	Missing Cases		Complete Cases		*P* *
	n/N	%	n/N	%	
***Socio-demographics***					
Age					<0.001
≤30 years	9,727/25,711	37.8	3,319/9,876	33.6	
>30 years	15,984/25,711	62.2	6,557/9,876	66.4	
Sex					0.258
Female	7,780/25,299	30.8	2,976/9,876	30.1	
Male	17,519/25,299	69.2	6,900/9,876	69.9	
Education					<0.001
Secondary or less	6,948/25,445	27.3	2,228/9,876	22.6	
Post-secondary	18,497/25,445	72.7	7,648/9,876	77.4	
Residence					<0.001
Rural	2,723/24,480	11.1	945/9,876	9.6	
Urban	21,757/24,480	88.9	8,931/9,876	90.4	
***Psychosocial factors***					
Very good or excellent health	13,656/25,166	54.3	5,455/9,876	55.2	0.100
Perceived effectiveness of handwashing to prevent COVID-19	8,418/27,187	31.0	3,336/9,876	33.8	<0.001
Personal action important for slowing COVID-19 spread	1,259/1,797	70.1	7,192/9,876	72.8	0.016
Knew someone diagnosed with COVID-19	1,165/1,267	92.0	9,150/9,876	92.7	0.372
Perceived norms: personal action is important for slowing COVID-19 spread	784/1,310	59.9	5,618/9,876	56.9	0.042
Practiced handwashing to prevent COVID-19, past week	1,844/2,164	85.2	8,916/9,876	90.3	<0.001

*P-values estimated using unweighted chi-square tests of association, comparing complete to missing cases.

**Table 3:** Unweighted descriptive sample statistics comparing complete cases (included in analysis) to those excluded from analysis due to missingness in Round 2.

	Missing Cases		Complete Cases		*P* *
	n/N	%	n/N	%	
***Socio-demographics***					
Age					<0.001
≤30 years	4,116/9,713	42.4	7,212/20,088	35.9	
31+ years	5,597/9,713	57.6	12,876/20,088	64.1	
Sex					<0.001
Female	2,810/9,481	29.6	5,547/20,088	27.6	
Male	6,671/9,481	70.4	14,541/20,088	72.4	
Education					<0.001
Secondary or less	3,338/9,447	35.3	5,685/20,088	28.3	
Post-secondary	6,109/9,447	64.7	14,403/20,088	71.7	
Residence					0.001
Rural	995/8,578	11.6	2,065/20,088	10.3	
Urban	7,583/8,578	88.4	18,023/20,088	89.7	
***Psychosocial factors***					
Very good or excellent health	4,865/9,260	52.5	10,701/20,088	53.3	0.242
Perceived effectiveness of handwashing to prevent COVID-19	4,211/11,023	38.2	9,009/20,088	44.9	<0.001
Personal action important for slowing COVID-19 spread	2,238/3,070	72.9	14,831/20,088	73.8	0.275
Knew someone diagnosed with COVID-19	1,611/1,781	90.5	18,285/20,088	91.0	0.421
Perceived norms: personal action is important for slowing COVID-19 spread	1,086/1,776	61.2	11.444/20,088	57.0	0.001
Practiced handwashing to prevent COVID-19, past week	2,984/3,879	76.9	16,680/20,008	83.0	<0.001

*P-values estimated using unweighted chi-square tests of association, comparing complete to missing cases.

Table 4 presents weighted descriptive sample statistics by survey round. While there were statistically significant differences in the socio-demographic characteristics of participants across rounds, point-prevalence differences were negligible. In general, in Rounds 1 and 2, participants tended to be older than 30 years old (59% and 58%, respectively), men (51% and 52%, respectively), and had at least a post-secondary education (77% and 72%, respectively). In addition, the majority (89%) of participants resided in urban areas across survey rounds.

**Table 4:** Description of study population (weighted percentages), by survey round (*N* = 29,964).

	Round 1 (*n* = 9,876)		Round 2 (*n* = 20,088)		*P* *
	n	%	n	%	
***Socio-demographics***					
Age					0.006
≤30 years	3,319	40.7	7,212	42.2	
>30 years	6,557	59.3	12,876	57.8	
Gender					0.016
Women	2,976	49.2	5,547	47.7	
Men	6,900	50.8	14,541	52.3	
Education					<0.001
Secondary or less	2,228	23.4	5,685	28.2	
Post-secondary	7,648	76.6	14,403	71.8	
Residence					0.168
Rural	945	10.6	2,065	11.1	
Urban	8,931	89.4	18,023	88.9	
***Ideational factors***					
Very good or excellent health	5,455	60.0	10,701	57.5	<0.001
Perceived effectiveness of handwashing to prevent COVID-19	7,192	74.4	14,831	75.3	0.093
Personal action important for slowing COVID-19 spread	9,150	92.1	18,285	91.1	0.003
Knew someone diagnosed with COVID-19	3,336	31.2	9,009	40.0	<0.001
Perceived norms: personal action is important for slowing COVID-19 spread	5,618	55.7	11,444	57.0	0.044

* *P*-values calculated using a design-adjusted F-statistic.

The distribution of most ideational factors was similar across survey rounds, despite some statistically significant differences. Specifically, more than half of study participants rated their overall health as very good or excellent at both rounds. The majority of all respondents across the survey rounds perceived handwashing to be effective in preventing COVID-19 (74% and 75% in Rounds 1 and 2, respectively), while about 90% of respondents perceived personal action was important to prevent COVID-19 (92% and 91% in Rounds 1 and 2, respectively). At Round 1, about a third (31%) of participants knew someone diagnosed with COVID-19. By Round 2, this increased to 40% (*p*<0.001). Over half of all respondents reported that most people in their community perceived personal action was important to prevent COVID-19 (56% and 57% in Rounds 1 and 2, respectively).

**Fig 2:**
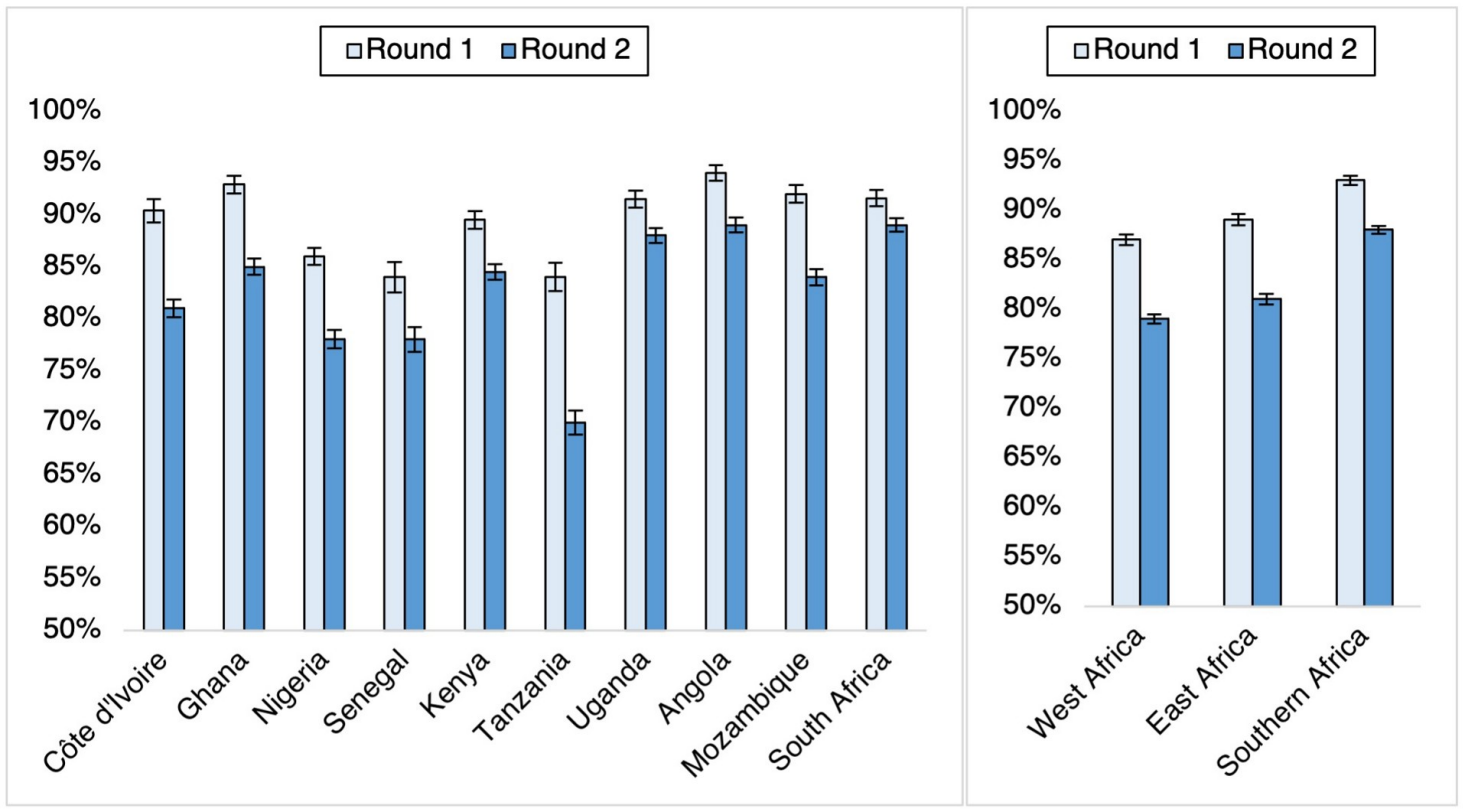
Prevalence (%) of handwashing in the past week to prevent COVID-19, by round (*N* = 29,964)*. *All calculated point-prevalence estimates were significantly (p<0.05) different between survey rounds, as determined by a design-adjusted F-statistic. Bars represent the upper and lower bounds of the 95% confidence intervals.

Fig 2 compares country-level prevalence of handwashing in the past week to prevent COVID-19 across survey rounds. At Round 1, the prevalence of handwashing in the past week ranged from 84% in Tanzania and Senegal to 94% in Angola. On a regional level, handwashing was highest in Southern Africa (93%) compared to East (89%) and West (87%) Africa. By Round 2, however, the prevalence of handwashing dropped across all countries, ranging from 70% in Tanzania to 89% in Angola and South Africa. On a regional level, handwashing remained highest in Southern Africa (88%) compared to East (81%) and West (79%) Africa. The decline in handwashing prevalence was most prominent in Tanzania (14 percentage points (pp) and Cote d’ Ivoire (9pp) but least in South Africa (3pp). On a regional level, the decline in handwashing by round 2 was least in Southern Africa (5pp) compared to East and West Africa (8pp). Country- and region-level declines in handwashing between Round 1 and Round 2 were statistically significant in all countries and regions (*p*<0.05).

**Fig 3:**
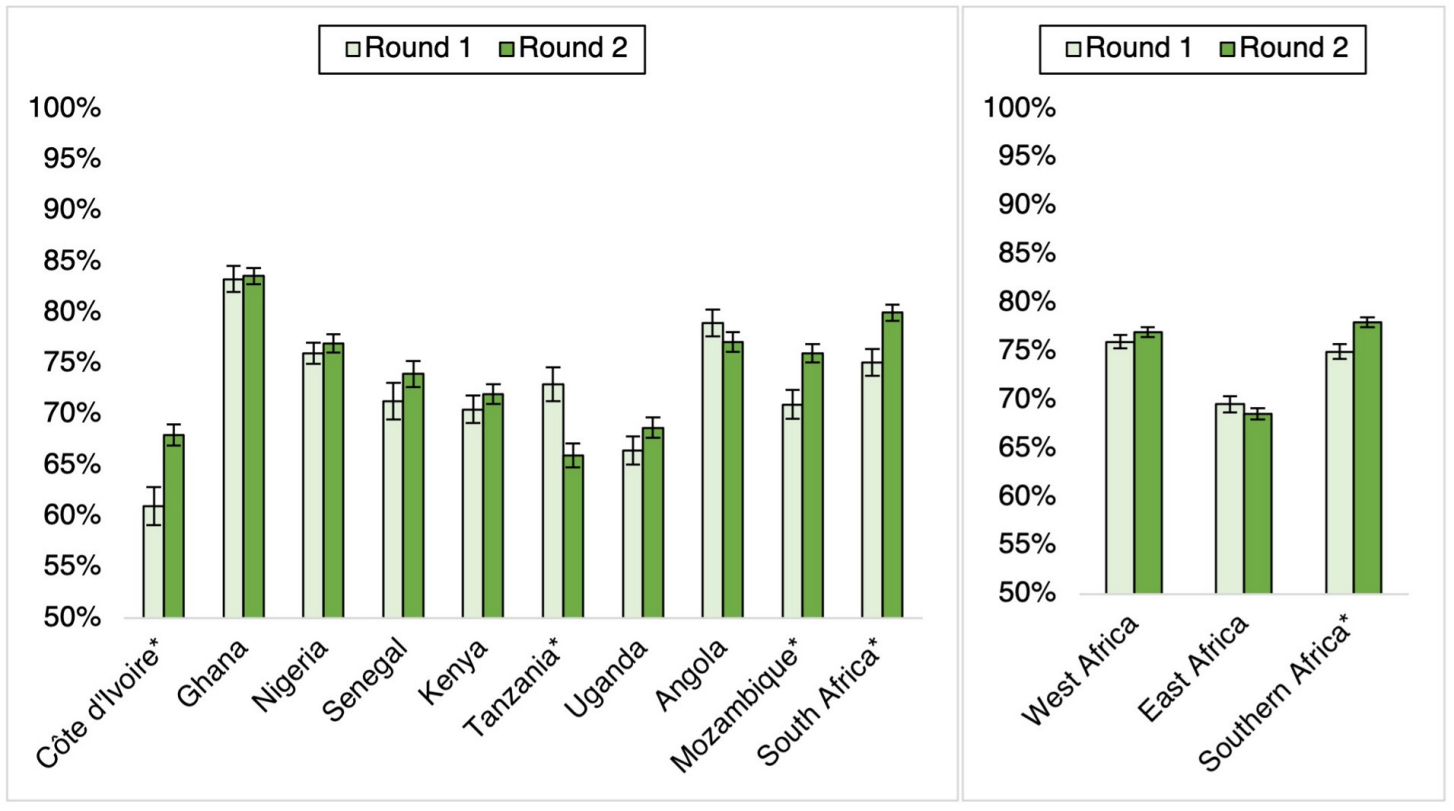
Prevalence (%) endorsing handwashing as effective for COVID-19 prevention, by round (*N* = 29,964)*. *Point-prevalence estimates were significantly (p<0.05) different between survey rounds, as determined by a design-adjusted F-statistic. Bars represent the upper and lower bounds of the 95% confidence intervals.

Trends in attitudes towards handwashing as a COVID-19 prevention strategy were mixed (see Fig 3). At Round 1, perceived effectiveness of handwashing ranged from 61% in Cote d’ Ivoire to 83% in Ghana. On a regional level, this belief was more commonly held in West (76%) and Southern (75%) compared to East (70%) Africa. The prevalence of perceived effectiveness of handwashing for COVID-19 prevention increased marginally from Round 1 to 2 in some countries. There was a marked increase in Cote d’ Ivoire (7pp), Mozambique and South Africa (5pp), while increases were less noteworthy in Senegal (3pp), Uganda (2pp), and Ghana, Nigeria, and Kenya (1pp). In contrast, declines in perceived effectiveness of handwashing were observed in Tanzania (7pp) and Angola (2pp). On a regional level, positive attitudes towards handwashing increased most in South Africa (3pp), compared to West (1pp increase) or East (1pp decrease) Africa.

Multivariable logistic regression estimates presented in Table 5 highlight socio-demographic and ideational factors associated with handwashing. Specifically, handwashing was higher among people who were older than 30 years (aOR = 1.25; 95%CI: 1.15–1.35) and had a post-secondary education (aOR = 1.62; 95%CI: 1.49–1.77). However, odds of handwashing were lower among men than women (aOR = 0.71; 95%CI: 0.64–0.78).

**Table 5:** Multivariable logistic estimates of handwashing by socio-demographic and ideational factors at Round 2 (*N* = 20,088)*.

	Adj. OR	95% CI	*P*
***Socio-demographics***			
Age			
≤30 years	1.00	*Ref*.	
>30 years	1.25	1.15–1.35	<0.001
Gender			
Women	1.00	*Ref*.	
Men	0.71	0.64–0.78	<0.001
Education			
Secondary or less	1.00	*Ref*.	
Post-secondary	1.62	1.49–1.77	<0.001
Residence			
Rural	1.00	*Ref*.	
Urban	0.96	0.85–1.09	0.543
***Ideational factors***			
Very good or excellent health	0.95	0.87–1.03	0.210
Perceived effectiveness of handwashing to prevent COVID-19	2.17	2.00–2.36	<0.001
Personal action important for slowing COVID-19 spread	2.93	2.60–3.31	<0.001
Knew someone diagnosed with COVID-19	1.28	1.18–1.40	<0.001
Perceived norms around personal action for slowing COVID-19 spread	0.87	0.79–0.95	0.001

*Logistic estimates adjusted for country (modeled as a fixed effect) and all covariates presented in the table.

Ideational factors associated with handwashing included perceived effectiveness of handwashing for COVID-19 prevention (aOR = 2.17; 95%CI: 2.00–2.36), perceiving personal action as important for slowing the spread of COVID-19 (aOR = 2.93; 95%CI: 2.60–3.31), and knowledge of someone diagnosed with COVID-19 (aOR = 1.28; 95%CI: 1.18–1.40). However, participants who perceived that people in their community thought that personal action was important to prevent COVID-19 had lower odds of handwashing (aOR = 0.81; 95%CI: 0.79–0.95).

**Fig 4:**
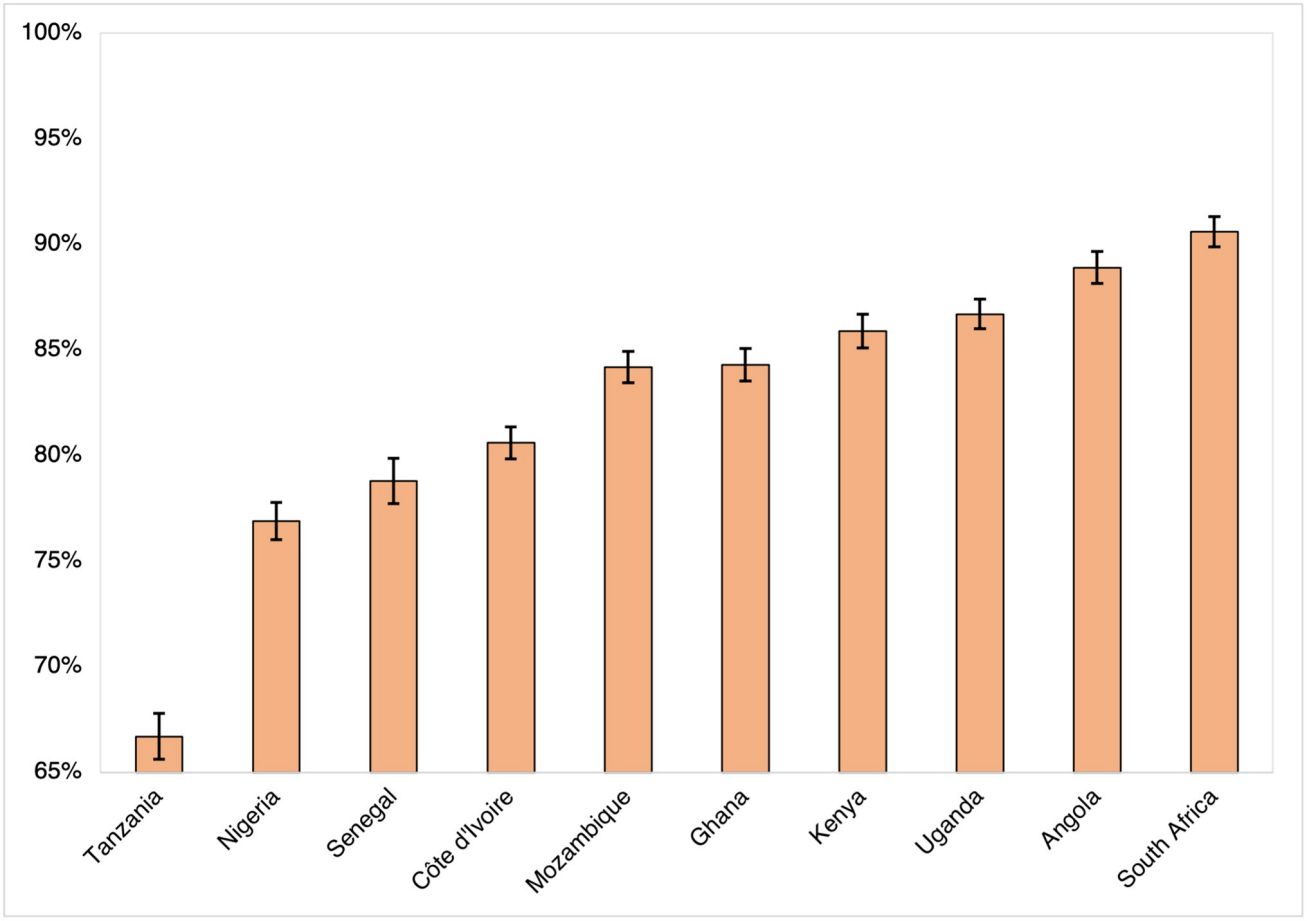
Adjusted marginal probability (%) of handwashing in the past week, by country (Round 2)*. *Marginal probabilities adjusted for age, sex, education, residence type, self-reported health status, vicarious COVID-19 experience, perceived efficacy of handwashing for COVID-19 prevention, endorsing personal action as important for COVID-19 prevention, and perceived norms around personal action for COVID-19 prevention.

Fig 4 shows the adjusted marginal probabilities of handwashing across study countries, controlling for socio-demographic and ideational factors described above. The adjusted marginal probability of handwashing was lowest in Tanzania (67%), Nigeria (77%), and Senegal (79%) but highest in Uganda (87%), Angola (89%), and South Africa (91%).

## Discussion

This study revealed relevant insights on handwashing during the COVID-19 pandemic in sub-Saharan Africa. Despite an increasing number of COVID-19 cases and deaths from July to November 2020, handwashing as a COVID-19 prevention strategy notably declined across ten countries. Factors associated with handwashing behaviours for COVID-19 prevention included older age, being a woman, higher levels of education, as well as key ideational factors such as knowing someone diagnosed with COVID-19 and positive perceptions of handwashing, and personal action, for COVID-19 mitigation, respectively.

There are a number of reasons posited for the decline in handwashing rates observed in this study. Observed reductions in handwashing may be attributed to uptake of other effective COVID-19 prevention behaviours, including wearing face masks and physical distancing [29]. Health communication messages for COVID-19 prevention may have emphasized the importance of wearing masks and maintaining physical distancing as priority COVID-19 prevention measures. Second, the protracted duration of the COVID-19 pandemic and associated restrictions may have led to pandemic fatigue, as people get tired of the pandemic measures and become less likely to follow public health practices or simply begin to drown out those messages [30]. Initial enthusiasm for taking action to address COVID-19 is replaced by feelings of exhaustion as the COVID-19 pandemic has become associated with psychological fatigue in other countries [31, 32]. Third, reduced perceived vulnerability to acquiring and/or perceived severity of infection may explain reduced handwashing rates in Africa, as witnessed in other contexts [33].

These findings reflect clear regional and country-level variation in handwashing as a COVID-19 preventive strategy. Given the critical role of handwashing as a strategy for preventing the spread of respiratory viruses, diarrheal diseases, and other outbreaks [3–6, 34], these differences indicate specific settings where social and behaviour change (SBC) interventions designed to support water, sanitation, and hygiene initiatives can have the greatest impact. Settings where access to handwashing stations is lowest (such as Angola or Kenya, for example) or where handwashing was most infrequent (such as Tanzania; see Figs 2 and 4) have the greatest opportunity for improvement but require distinct, adapted approaches to support handwashing effectively and sustainably. These differences underpin the COVID-19 pandemic’s heterogeneous impact on populations and countries across sub-Saharan Africa. This is reflected in the contrasting pandemic responses in study countries, from Tanzania–where communication about COVID-19 prevention was limited–to South Africa, where the government has actively implemented public health interventions to reduce transmission [35–37].

Differences in handwashing prevalence by age, gender, or education also speak to the opportunity to segment audiences for more targeted messaging on handwashing across settings. For example, participants who were younger, men, and less educated, respectively, had reduced odds of handwashing in the past week as compared to their older, female, and more educated counterparts. SBC interventions must develop specific, comprehensive messages designed to address the specific barriers to handwashing faced by these distinct audiences. Multivariable analysis showed that specific cognitive and social ideational factors such as perceived effectiveness of handwashing and perceived norms related to COVID-19 were significantly associated with handwashing practices across settings. Perceptions of community norms related to personal action as an important COVID-19 prevention measure were also associated with reduced odds of handwashing. This may be due to the fact that if people think others around them are taking precautions, they may feel less compelled to take precautions of their own. However, further research is needed to understand this finding as well as to expand understanding of the association between norms and context for handwashing.

In this context, SBC interventions should focus on addressing individuals’ perceptions related to handwashing. Evidence suggests that small-scale interventions that focus on ideational factors such as knowledge, attitudes, or social influence may influence handwashing with soap [38]. In light of these findings, efforts to scale up promising interventions are urgently needed. In addition, it is essential to understand the structural inequalities that restrict individuals’ access to handwashing stations or the water necessary for handwashing. Presence of handwashing stations varies significantly across sub-Saharan Africa, and water scarcity remains a significant barrier to following handwashing recommendations. For example, a qualitative study conducted in Zimbabwe explored the challenges to following the World Health Organization’s (WHO’s) Safe Hands campaign, demonstrating that to follow the recommendations would mean using an amount of water unavailable to many [39]. Furthermore, more frequent handwashing oftentimes meant increased frequency in water retrieval, which led to individuals, often women, being less able to engage in other preventive behaviours for COVID-19, like physical distancing, when waiting at the pump for water [39]. Existing and future handwashing campaigns for COVID-19 and other emerging infections must ensure that recommendations are relevant to the lived experiences of people residing in the communities where campaigns are implemented. SBC interventions should therefore couple a focus on ideational factors such as attitudes, perceptions, and norms with large-scale investments in infrastructure to strengthen the enabling environment for individuals to practice handwashing sustainably. There remains significant potential to advocate for greater investment in infrastructure and commitment to structural changes to improve access to water and handwashing at different stages of the COVID-19 pandemic [39–41].

This study is characterized by a number of strengths. First, it fills a gap in the paucity of data on handwashing behaviours in the context of COVID-19. A majority of publications to date are commentaries [25–27] and, therefore, do not contribute empirical evidence. Second, the online sampling and survey implementation strategy permitted inclusion of participants across multiple African nations, which facilitated comparisons in key handwashing indicators across countries with different SARS-CoV-2 epidemic profiles. Third, the study’s large sample size, which were weighted to reflect each country’s age/gender composition, generated insights that could approximate knowledge, attitudes, and behaviours in the general population relative to other smaller-scale studies implemented during the COVID-19 pandemic. Finally, the study’s theoretical orientation towards ideational factors facilitated careful measurement and selection of indicators that were proximal determinants of handwashing behaviour.

Nonetheless, findings from this study are subject to at least six limitations. First, due to the survey’s question block randomization schema, only participants with complete (non-missing) data on key variables were included in the analysis. Exclusion of participants with incomplete or missing data could, therefore, bias point estimates and effect sizes, as missing cases may be distinguishable from complete cases on various observable and unmeasured factors. Sensitivity analyses, nevertheless, revealed negligible differences between complete and missing cases (see Tables 2 and 3). Second, participants were active Facebook users, who may not represent the general population of each country. Given that Facebook users tend to have higher education, access to resources, and reside in urban areas relative to the general population [42], handwashing and other behavioural determinants are likely overestimated in this study. Analysis weights were, thus, used to correct for underlying discrepancies between the age and sex composition of the study population and the national populations from which participants were surveyed. Generalizing these results, nonetheless, to populations other than active Facebook users in the 10 countries at the time of survey implementation should be done cautiously, bearing in mind sampling techniques and resulting selection biases. Third, other dimensions of handwashing (i.e., correct use, frequency) were not captured or measured by the survey’s handwashing indicator. The specific context in which handwashing occurs, handwashing frequency, and compliance with recommended handwashing practices should be explicitly measured and examined in future research. Fourth, measures for handwashing and other ideational factors were self-reported and, therefore, subject to response and recall biases. Fifth, results from multivariable analysis did not account for COVID-19 case burdens or presence of SBC campaigns promoting handwashing for COVID-19 prevention, which could partially explain country-level variation in handwashing and produce biased estimates. SARS-CoV-2 surveillance heterogeneities across countries further complicate introduction of COVID-19 case burdens into analysis, as variable quality in estimates could introduce additional measurement error and further bias results. Self-reported vicarious experience with COVID-19 was, therefore, included in the analysis as an individual-level proxy of national COVID-19 case burdens. Lastly, sampling was done independently at each survey wave, producing serial cross-sectional samples of adult Facebook users at two different time points; observed changes in indicators over time could, therefore, reflect differences in the underlying study populations rather than temporal shifts in behaviour or ideational factors, as the data would suggest. Analysis weights were applied when calculating point estimates at each survey round, so results would be representative of the underlying age/sex distribution of each country’s national population and, therefore, more robust to any changes in the study sample’s composition at each round.

## Conclusion

In conclusion, this study sought to explore trends in and determinants of handwashing across sub-Saharan Africa. Among the mostly urban populations, handwashing rates declined despite increasing cases of COVID-19, with heterogeneity across settings. Factors associated with handwashing included older age, being a woman, higher education, knowing someone diagnosed with COVID-19, and perceptions regarding the effectiveness of handwashing and the importance of personal action, respectively, in preventing COVID-19. The findings suggest that COVID-19 prevention messages should continue stressing the importance of handwashing, in addition to face mask use and physical distancing. Furthermore, targeted interventions promoting behaviour change should employ approaches that address ideational factors, account for heterogeneity, as well as include complementary structural interventions.

## Supporting information

S1 Data Condensed dataset. (CSV)
